# Behavioural activation by mental health nurses for late-life depression in primary care: a randomized controlled trial

**DOI:** 10.1186/s12888-017-1388-x

**Published:** 2017-06-26

**Authors:** Noortje Janssen, Marcus J.H. Huibers, Peter Lucassen, Richard Oude Voshaar, Harm van Marwijk, Judith Bosmans, Mirjam Pijnappels, Jan Spijker, Gert-Jan Hendriks

**Affiliations:** 1 Behavioural Science Institute, Radboud University, Nijmegen, The Netherlands; 2 Department of Primary and Community Care, Radboud University Medical Centre Nijmegen, Nijmegen, The Netherlands; 3 Institute for Integrated Mental Health Care “Pro Persona, Nijmegen, The Netherlands; 4 Department of Clinical Psychology, VU University Amsterdam, Amsterdam, The Netherlands; 5 University Medical Center Groningen, Interdisciplinary Center for Psychopathology of Emotion regulation (ICPE), University of Groningen, Groningen, The Netherlands; 6 Centre for Primary Care, Institute for Population Health, University of Manchester, Manchester, UK; 7 Department of Health Sciences and EMGO Institute for Health and Care Research, Faculty of Earth and Life Sciences, VU university Amsterdam, Amsterdam, The Netherlands; 8 MOVE Research Institute Amsterdam, Faculty of Human Movement Sciences, VU University Amsterdam, Amsterdam, The Netherlands; 9 Department of Psychiatry, Radboud University Medical Centre Nijmegen, Nijmegen, The Netherlands

**Keywords:** Behavioural activation, Late-life depression, Primary care, Older adults, Depressive symptoms

## Abstract

**Background:**

Depressive symptoms are common in older adults. The effectiveness of pharmacological treatments and the availability of psychological treatments in primary care are limited. A behavioural approach to depression treatment might be beneficial to many older adults but such care is still largely unavailable. Behavioural Activation (BA) protocols are less complicated and more easy to train than other psychological therapies, making them very suitable for delivery by less specialised therapists. The recent introduction of the mental health nurse in primary care centres in the Netherlands has created major opportunities for improving the accessibility of psychological treatments for late-life depression in primary care. BA may thus address the needs of older patients while improving treatment outcome and lowering costs.The primary objective of this study is to compare the effectiveness and cost-effectiveness of BA in comparison with treatment as usual (TAU) for late-life depression in Dutch primary care. A secondary goal is to explore several potential mechanisms of change, as well as predictors and moderators of treatment outcome of BA for late-life depression.

**Methods/design:**

Cluster-randomised controlled multicentre trial with two parallel groups: a) behavioural activation, and b) treatment as usual, conducted in primary care centres with a follow-up of 52 weeks. The main inclusion criterion is a PHQ-9 score > 9. Patients are excluded from the trial in case of severe mental illness that requires specialized treatment, high suicide risk, drug and/or alcohol abuse, prior psychotherapy, change in dosage or type of prescribed antidepressants in the previous 12 weeks, or moderate to severe cognitive impairment. The intervention consists of 8 weekly 30-min BA sessions delivered by a trained mental health nurse.

**Discussion:**

We expect BA to be an effective and cost-effective treatment for late-life depression compared to TAU. BA delivered by mental health nurses could increase the availability and accessibility of non-pharmacological treatments for late-life depression in primary care.

**Trial registration:**

This study is retrospectively registered in the Dutch Clinical Trial Register NTR6013 on August 25th 2016.

## Background

With a point prevalence of 13,4% clinically relevant late-life depressive syndromes are common [1, 2]. A study in Dutch primary care practices found that major depression was prevalent in 13,7% and minor depression in 10,2% of the elderly patients [3]. However, most older patients with depression remain untreated [4]. Often older adults attribute their depressive symptoms to the ageing process or declining physical health [5–7], have low expectations about existing treatments [6], and/or feel adversity towards treatment in mental health clinics [8]. A large 3-year longitudinal cohort study on late-life depression in 32 Dutch primary care centres (PCC’s) showed the prognosis to be poor [9]. The direct economic costs of depression treatment as well as the indirect costs of depression associated with premature mortality and morbidity are high, as people with depression have a higher healthcare consumption than non depressed people [10–12]. The increasing ageing population in the Netherlands will raise healthcare costs and un(der)treated older patients with clinical depression will add to the burden.

Although late-life depression can be treated effectively, there is space for both improvement and development of alternative treatments [9, 13, 14]. Furthermore, especially in older adults, the use of psychoactive agents like antidepressants is associated with risks and adverse events, such as falls and adverse interactions with somatic medications [2, 15, 16]. Although older adults prefer psychological treatment over pharmacological treatment [1], only 3% of older patients with minor depression and 10% of older patients with major depressive disorder are referred to a mental health professional [15]. There are several barriers that can explain this underutilization of mental health services [17]. Older adults report a fear of stigmatization and have difficulties identifying their need for help, believing that their symptoms are normal [17]. Furthermore, there is a lack of psychotherapists trained in treating older adults and most facilities treating older patients for depression are located outside the PCCs [8]. Patients thus experience practical barriers such as transportation problems and are held back by the costs of treatment [13, 16, 17]. Many of these barriers could be overcome if an effective psychological treatment was available in primary care, one that acknowledges but does not medicalize the depressive feelings older adults experience. Behavioural activation (BA) might be that treatment.

BA aims to create a personal environment of positive reinforcement by increasing functional and pleasurable behaviour, and by decreasing avoidant and depressed behaviour [18, 19]. BA protocols are less complex, easier to train and to implement than other psychological therapies, making them very suitable for implementation in primary care, as studies from the UK have already shown for non-older adults [20, 21]. Currently, cognitive behavioural therapy (CBT) and interpersonal therapy (IPT) are the most frequently used treatments for depression, but research suggests that BA might be equally effective [22–25]. Recent introduction of the mental health nurse (MHN) in PCCs in the Netherlands has created major opportunities for the delivery of psychological treatments, such as BA, for late-life depression. The availability of BA in PCCs may improve accessibility and acceptability of psychological treatment for older patients [8]. BA may thus address the needs of older patients while improving treatment outcome and lowering costs [1, 26].

## Effectiveness of BA

Several meta-analyses [27, 28] as well as a recent RCT [24] among depressed patients found BA to be at least equivalent to CBT in the acute phase and at follow-up, and more effective than control conditions and some other psychological therapies for depression in adults. Moreover, MHN-delivered BA is both feasible, effective and cost-effective in managing depression in primary care [20, 21].

Meta-analyses with respect to BA treatment for late-life depression confirm its benefits for this age-group, although included studies were small and had low methodological quality [29–31]. Furthermore, two small observational studies showed that BA delivered by master level students and by social workers is feasible [32, 33].

Further evidence for the effectiveness of BA for late-life depression stems from a large RCT on care management for patients with late-life depression that suggest that activity scheduling, which exhibits similarities with BA, could be a major contributor to treatment gains [34]. This trial did not directly compare BA and TAU. In another RCT ‘collaborative care’ BA was the principal treatment compared to TAU for older adults with subclinical depressive symptoms. The study found a small to medium effect of collaborative care on depressive symptoms compared to TAU [35]. In both studies, BA was only a component of a new care management plan, so the direct effects of BA on depressive symptoms could not be determined.

Despite the growing evidence of the effectiveness of BA in managing depression, no methodologically sound and well-conducted studies have evaluated the effectiveness and cost-effectiveness of BA by MHNs as a stand-alone treatment for older depressed adults in primary care. BA, delivered by MHNs, could increase the availability and accessibility of non-pharmacological treatments for late-life depression in primary care.

## Mechanisms, predictors and moderators

As stated before, recent studies show that BA is non-inferior to CBT in adults [22]. Several other studies suggest that most psychological interventions for depression are equally effective, a phenomenon also referred to as the “dodo-bird verdict” [36, 37]. This doesn’t mean that all interventions are equally effective for all patients. Therefore we are specifically interested in the underlying mechanisms and predictors that can explain how therapeutic change comes about in BA.

BA theory suggests that changes in activation cause and precede the decline of depressive symptoms [18, 19, 38]. This has been demonstrated in a recent small scaled study that found that changes in activation preceded or co-occurred with changes in depression in adults, while in TAU this pattern was not found [39]. Another potential therapy-specific mechanism of BA is rumination. In BA, rumination is specifically targeted as a behavioural problem that can be actively tackled with behavioural interventions. BA attempts to change the act of rumination, thereby leaving more time for constructive and social behaviour, rather than challenging the content of the dysfunctional thoughts [18].

Several non-specific factors that are potentially related to treatment gain in depression as well as potential predictors of the (differential) effects are also investigated, such as loneliness, physical activity, cognitive functioning, therapeutic alliance, behavioural limitations, general psychopathology and treatment expectations [40–43].

## Objectives and hypotheses

In the current trial, we aim to evaluate the effectiveness and cost-effectiveness of BA, delivered by mental health nurses for older adults with moderate to severe depressive symptoms in PCCs. The study will investigate the effectiveness of BA in real life primary health care, with the aim to generalize results to primary care in the Netherlands and abroad.

**Fig. 1:**
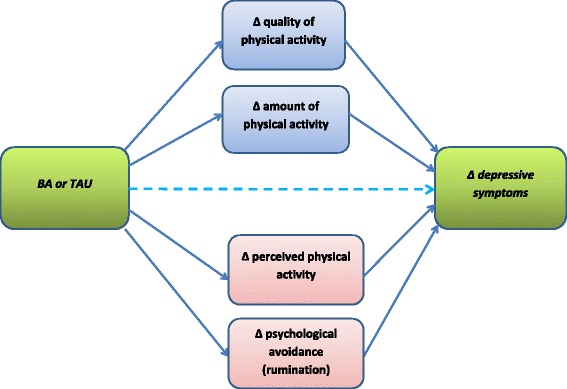
Theoretical framework for potential mechanisms of action

The second aim of this study is to investigate whether treatment gains in BA can be explained by treatment-specific factors based on BA-theory, such as the degree of activation, or non-specific factors, such as the therapeutic alliance or treatment expectations. An example of a possible mechanistic pathway that might explain the effectiveness of BA can be seen in Fig. 1.

The following main research questions were formulated:

1. What is the effectiveness of BA compared to TAU for older adults with clinically relevant depressive symptoms over the course of 12 months?
2. What is the cost-effectiveness of BA compared to TAU from a societal perspective for older adults with clinically relevant depressive symptoms, over the course of 12 months?
3. What are the mechanisms of change that account for the effectiveness of BA compared to TAU?
4. What are the predictors and moderators of outcome of BA compared to TAU?

In line with previous research, we hypothesize that BA is more effective and cost-effective than TAU [21–31].

## Methods

### Study design

The design of this study is a cluster randomized controlled multicentre trial with two parallel treatment groups with a follow up of 52 weeks. The treatment conditions are behavioural activation (BA), and treatment as usual (TAU). Data are collected at general practices in the South-Eastern region of the Netherlands. Only practices that employ a mental health nurse (MHN) are included. Currently, more than 80% of Dutch PCCs employ an MHN [44]. A list of currently participating PCCs can be found on http://beatdepressie.nl/voor-deelnemers/. We will report the study according to the SPIRIT guidelines [45]. The study has been approved by the local medical ethical committee of the Radboud University Medical Centre (CMO Arnhem-Nijmegen).

### Eligibility criteria

General practitioners and mental health nurses are responsible of the recruitment of participants. They include eligible patients during consultations (see procedures for details). We will include 200 older adults (≥65 years). All patients presenting with depressive symptoms or depression are asked to participate. Those who have a PHQ-9 score > 9 and have given informed consent will be included in the study. Patients will be excluded from the trial in the case of 1) current severe mental illness in need of specialized treatment, including bipolar disorder, obsessive-compulsive disorder, (history of) psychosis, high risk of suicide, drug and/or alcohol abuse, 2) psychotherapy in the previous 12 weeks or current treatment by a mental health specialist and 3) moderate to severe cognitive impairment. Patients with antidepressants are eligible provided that a stable dose has been maintained for at least 12 weeks before participating in the study.

### Interventions

BA aims to increase activation levels by helping patients engage in rewarding activities and reduce avoidance behaviour that diminishes distress in the short term but has adverse consequences in the long term [18]. Our BA protocol is based on the model developed by Martell and colleagues [18]. This original BA model consists of 12 to 24 one-hour sessions and is designed for patients referred to specialised mental healthcare institutions for treatment. The patients eligible for treatment in PCCs will be a more mixed population suffering from clinically relevant depressive symptoms although less severe than patients referred to specialist care. In studies on collaborative care for depressed older adults, BA-treatment by MHNs is delivered in 8 to 10 15–45 min sessions [34, 35]. We decided to adjust BA intensity to a level that would fit the contemporary Dutch model for MHN-delivered care in PCCs.

BA will be delivered in eight 30-min face-to-face sessions within eight weeks, with the first session lasting 45 min. All key elements of the original BA-protocol of Martell et al. are kept in, including a functional analysis, activity registration, activity scheduling and relapse prevention [46]. Prior to the start of the study, all MHNs of the BA group received a two-day training by licensed specialists on the use of the BA protocol. While treating patients, MHNs receive two-weekly online (skype) supervision in groups of three, led by one of the BA specialists that provided the training. MHNs fill in a session checklist for every patient to check for therapy adherence.

*Treatment as usual (TAU)* will be unrestricted and involves the GPs treatment options consistent with the guidelines of the Dutch College of GPs [47, 48]. At baseline GPs indicate their intended treatment, such as pharmacological treatment, psychological treatment by a MHN or a psychologist, or watchful waiting. At post-treatment GPs confirm whether the intended treatment plan was followed and which changes were made. Although activation is addressed as an advice to patients suffering from depression or depressive symptoms in the before mentioned guidelines, it is not offered as a systematic and protocolised intervention and therefore not comparable to the BA protocol adopted in our study.

### Procedure

Older adults that visit their GP with depressive symptoms, complaints of loneliness, reduced social functioning, medically unexplained symptoms, and/or frequent attenders without a clear somatic diagnosis will be screened for potential eligibility. If the GP or MHN suspects depressive symptoms or if the patient fulfils one of the aforementioned criteria, the GP or MHN will screen the patient with the first two questions of Mini International Neuropsychiatric Interview (M.I.N.I. 5.0.0.) [49] on depressive symptoms. If one or both of the questions is answered affirmatively, the GP will provide the patient with basic information about the study and will ask whether (s)he agrees to be contacted by our research assistant.

Eligible participants receive a letter with study information and are contacted by a research assistant to plan a baseline visit, that takes place approximately one week after they’ve received the letter. This will allow them to make an informed decision about their participation. At the baseline visit a research assistant will visit the patient at home to assess whether (s)he fulfils the inclusion criteria and is willing to participate. During this visit, also several cognitive tests will be performed, and the presence of depressive disorder will be established with a semi-structured clinical interview M.I.N.I. 5.0.0 [49]. After the baseline meeting, the patient will receive the online or paper-pencil baseline questionnaire, which they will be asked to complete within the following week. Furthermore, the patient will wear an accelerometer for one week. Approximately one week after the baseline meeting the BA- or TAU-treatment is started.

During the treatment phase (baseline to 9 weeks), patients in both research conditions will receive a questionnaire every two to four weeks at home. BA patients also fill in a short questionnaire during the weekly sessions with the MHN. Nine weeks after the baseline assessment, patients will receive a call from a research assistant for the post-treatment assessment consisting of two cognitive tests, as well as a short interview to establish the presence of depressive disorder. After this, patients will wear the accelerometer for another week, and complete the post-treatment questionnaire within that week.

In the follow-up period (3 to 12 months after baseline), patients will receive a follow-up questionnaire every three months. After twelve months the last follow-up home-visit by a research assistant will be planned.

For a detailed schedule of all measurements per time-point, see Table 1.

**Table 1:** Participant timeline

		Treatment phase (weekly)									Follow up (3 monthly)			
Measures^a^	-1	0	1	2	3	4	5	6	7	8	3	6	9	12
Clinical outcome measures														
Depression (PHQ-9)^b^		•	•	•	•	•	•	•	•	•	•	•	•	•
Mini 5.0 (2 questions)	•													
MINI 5.0 interview (baseline complete, T8 only depression)		•								•				
Cognitive (MoCA)		•												•
Depression (QID-S)		•								•	•	•	•	•
Well- being & mood^c^ (visual analogue scale)			•	•	•	•	•	•	•	•				
Psychopathology *(BSI-SF)*		•								•				•
Limitations (WHODAS)		•								•				•
Process BA-specific														
Behavioural activity (BADS)		•		•		•				•	•	•	•	•
Physical activity (accelerometer)		•								•				
Ruminative brooding (RRS)		•		•		•				•	•	•	•	•
Process general														
Loneliness (S&ES)		•				•				•	•	•	•	•
Therapeutic alliance (SRS)^c^			•	•	•	•	•	•	•	•				
Cognitive (STROOP & DSST)		•								•				•
Expectancy (Expectancy and credibility list)^c^		•												
Cost effectiveness														
TIC-P		•									•	•	•	•
EQ-5D		•								•	•	•	•	•

^a^− 1 = screening by GP, 0 = baseline measurement by research assistant, 8 = post-treatment measurement by research assistant ^b^administered weekly in BA and two-weekly in TAU. ^c^only administered in BA

## Outcomes

### Clinical outcome measures

#### Depressive symptoms

The Quick Inventory of Depressive Symptomatology (QIDS-SR) will be used as the primary outcome measure of depressive severity. The QIDS-SR is a 16-item self-report instrument assessing depressive symptoms during the last two weeks. It can be administered in 5–7 min and has good psychometric properties [48].

The Patient Health Questionnaire- 9 (PHQ-9) is a 9-item self-report instrument assessing depressive symptoms during the last week. It will be used as a secondary outcome measure and to assess changes in depressive symptoms over the course of treatment. The psychometric properties of the PHQ-9 are good [50, 51]. Patients scoring PHQ-9 > 9 are classified as having clinically relevant depressive symptoms [52].

#### Depressive disorder and psychiatric comorbidity

To distinguish patients with a major depressive disorder (MDD) from patients with only clinically relevant symptoms a research assistant will administer the complete Mini International Neuropsychiatric Interview (M.I.N.I. 5.0.0). Furthermore, this instrument assesses psychiatric co-morbidity. The M.I.N.I. 5.0.0 is a short diagnostic interview that assesses current psychological disorders, based on DSM-IV diagnostic criteria. The reliability and validity of the M.I.N.I are good [49]. The M.I.N.I. can be administered in about 15 min [53].

### Economic evaluation

#### Quality of life

Quality of life will be evaluated using the five level version of the self-report EuroQol questionnaire (EQ-5D-5 L) [54]. The EQ-5D-5 L consists of five health-state dimensions on which respondents indicate their perception of their health-related well-being. Each dimension is rated as causing ‘no problems’, ‘slight problems’, ‘some problems’, ‘moderate problems’ or ‘extreme problems’. The Dutch EQ-5D tariff will be used to calculate Quality-Adjusted Life-Years (QALYs) [55]. The EQ-5D-5 L appears to be a valid extension of the reliable and valid EQ-5D-3 L [56].

#### Health care utilisation

Healthcare consumption and productivity losses will be recorded every three months in both study conditions using a modified version of the *Trimbos and iMTA questionnaire on Costs associated with Psychiatric illness* (TiC-P) [57]. Healthcare costs include the number of consultations with health care providers, medication and admissions to hospitals. Lost productivity costs are defined as the productivity lost due to absenteeism from (unpaid) work and reduced efficiency at work.

### Process and predictor variables

#### Psychopathology

Psychopathology will be measured using the Brief Symptom Inventory-18 (BSI-18) [58, 59]. The BSI-18 has three subscales: depression, anxiety and somatic symptoms, that together make up the Global severity Index (GSI) of psychological distress. Patients rate on a five-point Likert scale how much they have been bothered by each symptom in the last 7 days. The BSI-18 is a valid and reliable measure for psychological distress, and can detect depression and anxiety based on a DSM-IV classification in older adults [60].

#### Behavioural limitations

To assess the behavioural limitations that patient experience in daily life, The World Health Organization Disability Assessment Schedule 2.0 (WHODAS, short version) will be used [61]. The short version of the instrument has 12 items on a 5-point Likert scale. The instrument has proven to be able to measure the impact of health conditions such as depression on daily life and can monitor the effectiveness of interventions [62].

#### Cognitive functioning

To test baseline cognitive functioning the Montréal Cognitive Assessment Scale (MoCA) is used. The MoCA is a short cognitive screening tool that tests a wide range of cognitive functions [63]. The tool has a high sensitivity as well as specificity for detecting mild cognitive impairment, and is especially interesting for the population of depressed elderly because it measures executive functioning [63].

#### Behavioural activation

To measure perceived active behaviour in both BA and TAU conditions the Behavioural Activation for Depression Scale (BADS) can be used. The BADS is a 25-item self-report instrument measuring behavioural activation [64]. Four domains of behavioural activation are distinguished: Activation, avoidance/rumination, work/school impairment and social impairment. The work/school domain may seem less relevant for our population, but the questions are formulated in such a way that they could easily be applied to general responsibilities like household chores and volunteering. The psychometric properties of the BADS are good [64].

#### Rumination

To assess the extent of depressive brooding, the brooding subscale of the Rumination Response Scale (RRS) will be used [65–67]. The brooding scale consists of 5 questions on a 4-point Likert-scale. The reliability, and convergent and discriminant validity of the brooding scale are appropriate, and it predicts depressive symptoms prospectively [66].

#### Physical activity

The expected influence of BA on physical activity of depressed participants can be objectively registered with an accelerometer (DYnaPort MoveMonitor). Daily physical activities will be measured over a 1-week period at the start of the intervention and 10 weeks after baseline. Participants will be asked to wear a sensor at their lower back, by use of an elastic belt around their waist. The amount of daily activities will be determined, based on the MoveMonitor algorithms, which classifies the acceleration data into 4 types of activity: sitting, lying, standing and locomotion. Also, non-wearing time per day will be detected. Subsequently, the duration of locomotion, lying, sitting, and standing, movement intensity, the number of locomotion bouts and transitions to standing, and the median and maximum duration of locomotion will be calculated per day [68].

The quality of daily activities will be determined as the median values of gait characteristics, calculated over all episodes of locomotion during the week according to [69]. These characteristics include local divergence exponent, inter-stride variability, and entropy. In addition, we will determine the complexity of physical activity, based on the concept of ‘barcoding’ [70]. Such a barcode reflects different states of physical activity, based on features such as type, intensity, and duration of lying, sitting, standing and locomotion, and is quantified by structural complexity metrics and sample entropy. The accelerometer can be worn at all times except during water activities such as swimming or showering. For a detailed schedule of the timing of several measures, see Table 1.

#### Loneliness

The De Jong Gierveld Short Scale for emotional and social loneliness, a 6-item self-report measure will be used [71]. Patients answer three questions about social loneliness, characterized by a smaller number of friends than is considered desirable, and three questions about emotional loneliness, characterized by a lack of intimacy in current relationships, on a 6-point scale. The sum of both subscales indicates overall loneliness. The Dutch version of this short form has good psychometric properties [71].

#### Therapeutic alliance

To measure therapeutic alliance, the Session Rating Scale (SRS) is the instrument of choice. It consists of 4 visual analogue scales and aims to measure the self-reported therapeutic alliance by the patient [72]. The psychometric properties of this instrument are satisfactory and the feasibility in clinical practice is found to be good [72].

#### Psychomotor performance

With the Digit Symbol Substitution Test (DSST) the degeneration or improvement of psychomotor performance can be measured [73]. The DSST is pencil and paper test in which the patient is given a combination of numbers and matching symbols and a test section with numbers and empty boxes. The patient is asked to fill as many empty boxes as possible with a symbol matching each number within 90 s.

#### Executive functioning

To measure whether behavioural activation is effective regardless of the level of executive functioning of the patient, the Stroop-task will be used [74, 75]. Patients will be presented with three different sheets of words and/or colours. The first sheet contains colour names in black ink. Patients are asked to read the colour names. The second sheet consists of squares printed in different colours. Patients are asked to name the colours on the sheet. The last sheet contains the words “red”, “green” and “blue” that are printed in sometimes the same (e.g. the word red in red ink) and sometimes a different colour ink. The task is to name the colour of the ink in which the word is printed as quickly as possible. The increase in time taken to perform the latter task compared with the basic task is referred to as “the Stroop interference effect”.

#### Treatment expectations

To asses treatment expectations, the credibility & expectancy questionnaire will be used [76]. The questionnaire consists of six questions that are assessed on a 9 point Likert-scale. The questionnaire appears to have high internal consistency within each factor and good test–retest reliability [77]. Expectancy measures will not be used to ensure initial equivalence between two compared treatments, but to measure individual differences in expectancy within the BA group.

### Sample size

Comparing the effects BA for depression to a waiting list condition, Cuijpers et al. reported an effect size of 0.9 in a meta-analysis [27]. Since we will be comparing BA to a more active treatment condition (TAU) in depressed older patients, we estimate the effect size for depression outcome of BA versus TAU to be 0.50, a modest but clinically relevant value. Based on this estimate (alpha = 0.05, power (beta) = 0.80, 2-tailed test) and data clustering within PCCs (ICC of 0.05 and a cluster size of *n* = 6) 100 patients are required per group with two groups of 14 clusters (based on 14 potential patients per PCC and a 50% refusal and a 20% dropout rate).

### Recruitment

Several general practices in the South-Eastern region of the Netherlands are recruited to participate in the study. General practitioners and mental health nurses are responsible of the recruitment of participants.

### Assignment of interventions

PCCs are the units of randomization. PCCs that employ the same MHN will be in the same treatment condition, preventing contamination. An independent statistician will use pairwise matching to randomize the PCCs, taking into account the amount of MHNs in a PCC, as well as the sex of the MHN and the size of the patient population. In half of the PCCs the MHNs are trained to give BA-treatment while the other half will treat patients as usual. GPs, patients and MHNs are not blinded to treatment condition because PCCs are the units of randomisation, but the research assistants that assess depression after treatment are blind to condition.

### Data management

Data are handled confidentially as each patient is coded with a number. The link between the patient code and patient information is preserved in a digital document that will be protected by a password. This document is only available to the principal investigator, the executive researcher and the two research assistants. Coded patient data are kept in a folder that is stored within a different section of the RadboudUMC database than the source data. Informed consent forms are kept in a locked closet, separated from the paper and pencil questionnaires that also will be archived in a locked closet. Digital data are stored in Castor EDC, a program with a built in audit trail. Patients will send the filled in questionnaires by mail or e-mail. A research assistant will call patients when there is missing information in a questionnaire, or when a questionnaire is not returned in time. When a patient does not want to continue in the study, all the information up until then is used in the analyses but no additional information is collected.

### Statistical analyses

#### Clinical outcomes

Data analyses include intention-to-treat analyses and per-protocol analyses. The relative effectiveness of behavioural activation and treatment as usual will be analysed using mixed linear regression modelling. In addition, we will determine the proportion of patients that show reliable and clinically significant improvement on the outcome measures, based on the model of Jacobson and Truax [78, 79]. Relapse (episode of MDD after remission) and recurrence (episode of MDD after recovery) will be assessed in the course of follow-up (6, and 12 months) using survival analysis (Cox proportional hazards regression). Intention to treat analyses as well as per protocol analyses will be conducted.

#### Economic evaluation

The aim of the economic evaluation is to relate the difference in societal costs between BA and TAU to the difference in clinical effects. Both a cost-effectiveness and a cost-utility analysis will be performed with a time horizon of 12 months. The analysis will be performed in accordance with the intention-to-treat principle. Missing cost and effect data will be imputed using multiple imputation. Bivariate regression models will be used to estimate cost and effect differences between the groups while adjusting for relevant confounders. Incremental cost-effectiveness ratios (ICERs) will be calculated by dividing the difference in mean total costs between the two treatment groups by the difference in mean effects. Bias-corrected accelerated bootstrapping with 5000 replications will be used to estimate 95% confidence intervals around cost and effect differences, and uncertainty around the ICERs. Uncertainty surrounding ICERs will be graphically presented on cost-effectiveness planes. Cost-effectiveness acceptability curves will also be estimated showing the probability that BA is cost-effective in comparison with TAU for a range of different ceiling ratios thereby showing decision uncertainty [80].

#### Process variables and predictors

To identify mechanisms of change and the magnitude of the factors involved, both multilevel mediation models and structural equation models (using path analysis, such as Latent Difference Score (LDS) Models) will be used. Predictors (prognostic factors) and moderators (prescriptive factors) of outcome will be identified using mixed regression, building on the effectiveness models.

### Monitoring

Given the negligible risks of this study, the monitor is an independent researcher of the Primary and Community Care department (ELG) of the Radboud University Medical Centre.

### Harms

We will report all Serious Adverse Events (SAEs) to the sponsor without undue delay after obtaining knowledge of the events. We will report the SAEs through the web portal *ToetsingOnline* to the accredited Medical Ethics Committee that approved the protocol, within 7 days of first knowledge for SAEs that result in death or are life threatening followed by a period of maximum of 8 days to complete the initial preliminary report. All other

SAEs will be reported within a period of maximum 15 days after the sponsor has first knowledge of the serious adverse events.

## Discussion

The aim of this study is to investigate whether BA will be more (cost-)effective than TAU for older adults in primary care. Although BA has been proven to be an effective treatment for depression in adults, the effectiveness of BA delivered by MHNs in older adults, as well as the possible mechanisms of change are not yet clear. The results of the study can be used to improve the quality and availability of treatments for depressed older adults in primary care.

The main strength of this study is that procedures followed in this effectiveness trial closely match the daily practice of PCCs. Therefore the results can be easily generalized to other PCCs. Furthermore, our BA protocol is adapted to a primary care setting, is very easy to train, and therefore easily implemented in other PCCs throughout the Netherlands and abroad.

However, this study also has some limitations that will be taken into account. The main limitation derives from the fact that patients as well as some research assistants, GPs and MHNs are not blinded to patient allocation, due to cluster randomisation. This might influence their judgement of severity of depression of the patients. To prevent biased opinions on whether or not patients (still) have MDD post-treatment, the clinical interview will be performed by an independent research assistant that will be blinded to patient allocation.

Furthermore, we realize that the recruitment of patients in primary care could be challenging [81]. We are aware of the fact that Lasagna’s law, that states that medical investigators tend to overestimate the number of patients available, holds in Dutch primary care research [82]. It might be difficult for GPs to inform depressed older adults about the study in their 10 min sessions. Therefore the only task GPs will have, is to ask patients whether they may be contacted by a research assistant to provide them with more information. From there on, the research assistant will take over.

Lastly, older adults might have difficulties filling in questionnaires due to physical problems such as visual impairments, concentration problems or unfamiliarity with psychological questionnaires. Therefore an independent research assistant, blinded to patient allocation, is available when needed to help patients at home or by phone with the questionnaires.

If proven effective, BA can be a valuable addition to current guidelines for the treatment of older adults with moderate to severe depressive symptoms in primary care.

## Abbreviations

BA: Behavioural Activation
BADS: Behavioural Activation for Depression Scale
BIA: Budget Impact Analysis
BSI − 18: Brief Symptom Inventory-18
CBT: Cognitive Behavioural Therapy
CEA: Cost effectiveness Analysis
DSST: Digit Symbol Substitution Test
EuroQol-5D-5 L: European Quality of Life Self-report Questionnaire
GP: General Practitioner
IPT: InterPersonal Therapy
M.I.N.I. 5.0.0: Mini International Neuropsychiatric Interview
MDD: Major Depressive Disorder
MHN: Mental Health Nurse
MoCA: Montréal Cognitive Assessment scale
PCC: Primary Care Centre
PHQ-9: Patient Health Questionnaire- 9
QALYs: Quality-Adjusted Life-Years
QIDS-SR: Quick Inventory of Depressive Symptomatology
RCT: Randomised Controlled Trial
RRS: Rumination Response Scale
SRS: Session Rating Scale
TAU: Treatment As Usual
TiC-P: Trimbos and iMTA questionnaire on Costs associated with Psychiatric illness
WHODAS: World Health Organization Disability Assessment Schedule 2.0
